# Increased age and the volume of intraoperative fluid administered predict urinary retention after elective inguinal herniorrhaphy

**DOI:** 10.1186/s13741-024-00446-z

**Published:** 2024-08-19

**Authors:** Jin-Ming Wu, Chi-Chuan Yeh, Nathan Wei, Hsing-Hua Tsai, Shang-Ming Tseng, Kuang-Cheng Chan, Kuo-Hsin Chen

**Affiliations:** 1 Department of Surgery, National Taiwan University Hospital and National Taiwan University College of Medicine, 7 Chung-Shan South Rd., Taipei, 10002 Taiwan, ROC; 2 Department of Anesthesiology, National Taiwan University Hospital and National Taiwan University College of Medicine, 7 Chung-Shan South Rd., Taipei, 10002 Taiwan, ROC; 3 Department of Surgery, Far Eastern Memorial Hospital, New Taipei City, Taiwan, ROC; 4 Division of Electrical Engineering, Yuan Ze University, Taoyuan, Taiwan, ROC

**Keywords:** Inguinal hernia, Urinary retention, Hernia repair, Age, Intraoperative fluid

## Abstract

**Background:**

Inguinal hernia repair (IHR) is a common surgical procedure worldwide. Although IHR can be performed by the minimally invasive method, which accelerates recovery, postoperative urinary retention (POUR) remains a common complication that significantly impacts patients. Thus, it is essential to identify the risk factors associated with POUR to diminish its negative impact.

**Methods:**

We conducted a single-center retrospective review of elective IHR from 2018 to 2021. POUR was defined as the postoperative use of straight catheter or placement of an indwelling catheter to relieve the symptoms. Adjusted multivariate regression analysis was performed to address the associations of clinicodemographic, surgical, and intraoperative factors with POUR.

**Results:**

A total of 946 subjects were included in the analysis after excluding cases of emergent surgery, recurrent hernia, or concomitant operations. The median age was 68.4 years, and 92.0% of the patients were male. Twenty-three (2.4%) patients developed POUR. In univariate analysis, POUR in comparison with non-POUR was significantly associated with increased age (72.2 versus 68.3 years, *P* = 0.012), a greater volume of intraoperative fluid administered (500 versus 400 ml, *P* = 0.040), and the diagnosis with benign prostate hypertrophy (34.8% versus 16.9%, *P* = 0.025). In the multivariate model, both increased age (odds ratio [OR] 1.04, 95% CI 1.01–1.08; *P* = 0.049) and a greater volume of intraoperative fluid administered (OR 1.12 per 100-mL increase, 95% CI 1.01–1.27; *P* = 0.047) were significantly associated with the occurrence of POUR.

**Conclusions:**

We found that increased age and a greater volume of intraoperative fluid administered were significantly associated with the occurrence of POUR. Limiting the administration of intraoperative fluid may prevent POUR. From the perspective of practical implications, specific guidelines or clinical pathways should be implemented for fluid management and patient assessment.

## Introduction

Inguinal hernia repair (IHR) is one of the most common surgical procedures and is performed in more than 20 million patients worldwide each year (Kockerling and Simons 2018). The lifetime incidence rates of inguinal hernia are higher in men (25–40%) than in women (3–6%) (Kingsnorth and LeBlanc 2003), and surgical repair is the only curative treatment. In the modern era, minimally invasive techniques, such as laparoscopy and robotic surgery, enhance recovery compared to the traditional open method (Kockerling and Simons 2018; Qabbani et al. 2021; Kohno et al. 2022; Chai et al. 2022). Although the aforementioned advancements improve surgical outcomes, some complications following IHR are still relatively common.

Postoperative urinary retention (POUR; defined as the need for postoperative urinary catheterization due to the failure to void spontaneously) is a well-recognized complication of IHR (Blair et al. 2017). Although IHR-related POUR is often labeled a minor complication, it can significantly impact the quality of life of patients, provoking discomfort and restlessness, prolonging the length of hospital stay, and increasing medical costs (Croghan et al. 2023; Shukla et al. 2023). Previous studies have reported that POUR accounts for nearly 10% of ambulatory failures and is the main reason for readmission (Drissi et al. 2020). If POUR is not managed in a timely manner, collagen deposition between the smooth muscle fibers of the detrusor can occur, which reduces the contractile function of the urinary bladder and causes chronic impairment of emptying ability (Clancy et al. 2018).

Several studies have identified risk factors for POUR following IHR, including patient demographics (advanced age, male sex, obesity, and history of prostate hypertrophy) and perioperative variables (anesthesia method and surgical technique) (Aleman et al. 2021; Baldini et al. 2009; Darrah et al. 2009; Ferahman et al. 2021; Jensen et al. 2002; Sanjay and Woodward 2007). Notably, these studies reached inconclusive and sometimes contradictory results because of the lack of a standardized definition of POUR and heterogeneity in patient characteristics (Drissi et al. 2020). Furthermore, these studies are mainly based on small sample size, and details regarding the intraoperative process are frequently not specified, which precludes a more comprehensive analysis of the multifactorial nature of POUR following IHR. A comprehensive analysis of associated risk factors would inform clinical strategies to enhance patient recovery and improve perioperative quality. Therefore, the aim of this study was to investigate the intraoperative risk factors for POUR following IHR. By identifying these risk factors, we aim to inform clinical strategies to enhance patient recovery and improve perioperative quality.

## Methods

This study was a retrospective review of all consecutive patients who underwent either open or laparoscopic IHR between January 2018 and December 2021 in one academic medical center. The data retrieved from the research database were encrypted and anonymized, and the relevant institutional review board approved this study (National University Hospital; 202008052RINC) in accordance with the Declaration of Helsinki. The main drawback of a retrospective study is the potential for bias and confounding. Issues include selection bias, information bias, recall bias, unaccounted confounding variables, difficulty in establishing temporal relationships, and limited control over data quality, which can lead to inaccurate or skewed results. These drawbacks of the nature of one retrospective study are addresses in the limitation section.

All patients aged 20 years or older undergoing elective IHR were eligible for inclusion in the study. Patients undergoing elective IHR for recurrent hernia, emergency IHR, any concomitant operation, or those with an indwelling urinary catheter or urinary diversion were excluded.

The definition of POUR was the postoperative use of straight catheters or placement of an indwelling catheter to relieve the symptoms (Blair et al. 2017).

Patient data included age, sex, body mass index, and marital status. The original weighted Charlson comorbidity index (CCI) score was used to represent the comorbidity burden, and comorbidities were documented according to electronic medical records before IHR (Quan et al. 2011; Wu et al. 2019). Postoperative complications were considered major if the Clavien–Dindo Classification grade was III or above (Dindo et al. 2004). Anesthesia and intraoperative data were collected from the same electronic database. The potency of anesthetics was divided into three categories: short-acting, intermediate-acting, and long-acting (Mulroy et al. 2002; Becker and Reed 2012). The volume of intraoperative fluid administered and the intraoperative use of vasopressors were also retrieved.

All IHR procedures were performed as an inpatient surgery, and the patients were discharged the day after IHR if the course was uneventful according to the clinical pathway of the Diagnosis-Related Group of the Taiwan National Healthcare System.

### Statistical analysis

All statistical analyses were performed using Stata/SE 15.0 (StataCorp, College Station, Texas, USA). Data are presented as the median (interquartile range [IQR]), number (percentage), or odds ratio (OR) and 95% confidence interval (CI). The dependent variable (POUR) was coded as a categorical variable (yes or no).

The χ^2^ test or Fisher’s exact test (if expected frequencies were < 5) was used to compare categorical variables between the POUR and non-POUR groups. These tests are appropriate for determining the association between two categorical variables.

The nonparametric Mann–Whitney *U* test was used to compare continuous variables between the two groups due to the non-normal distribution of these variables. This test is suitable for comparing medians between two independent groups when the assumption of normality is violated. Furthermore, a multivariate logistic regression analysis was performed to assess the correlation between the occurrence of POUR and the univariate factors identified within a confidence interval of 90%. This method allows for the adjustment of potential confounding variables and provides adjusted odds ratios (OR) with 95% confidence intervals (CI) to quantify the strength of the associations.

In all hypothesis testing, the null hypothesis was rejected with a type I error rate (α) of less than 0.05. All statistics were 2-tailed, and *P* < 0.05 was considered statistically significant.

## Results

During the study period, there were 1002 IHR patients. Among the 946 subjects meeting our inclusion and exclusion criteria during the study period, the median age was 68.4 years, and 92.0% of the patients were male (Table 1). Twenty-three (2.4%) developed POUR (the POUR group). The numbers of subjects with an American Society of Anesthesiologists (ASA) score ≥ 3 and a CCI score > 2 were 387 (40.9%) and 271 (28.6%), respectively. A total of 66 cases (7.0%) received bilateral IHR. Only one (0.1%) case developed major complication rate, and there was no mortality.

**Table 1:** Demographic and clinical characteristics of the study population (*N* = 946)

Factor	Number (%) or median (IQR)
Age, years	68.4 (59.7, 74.8)
Sex	
Female	76 (8.0%)
Male	870 (92.0%)
Body mass index, kg/m^2^	23.2 (21.2, 25.1)
Charlson comorbidity index score	
≤ 2	675 (71.4%)
> 2	271 (28.6%)
American Society of Anesthesiologists physical status	
< 3	559 (59.1%)
≥ 3	387 (40.9%)
Minimally invasive surgery	14 (1.5%)
Bilateral repair	
No	880 (93.0%)
Yes	66 (7.0%)
Spinal anesthesia	239 (25.3%)
Volume of intraoperative fluid	400.0 (200.0, 500.0)
Major complication	1 (0.1%)
Length of hospital stay	3.0 (3.0, 3.0)
Surgery duration (minutes)	69.0 (53.0, 86.0)
Blood loss (ml)	0.0 (0.0, 0.0)
Postoperative urinary retention	23 (2.4%)

In the univariate model (Table 2), the POUR group had a significantly higher median age than the non-POUR group (72.2 versus 68.3 years, *P* = 0.012). Furthermore, the median volume of intraoperative fluid administered in the POUR group was significantly greater than that in the non-POUR group (500 versus 400 ml, *P* = 0.040). There was a significantly higher proportion of BHP in the POUR group than in the non-POUR group (34.8% versus 16.9%, *P* = 0.025). No significant differences were observed in the proportions of sex, body mass index, minimally invasive surgery, bilateral repair, spinal anesthesia, or type of anesthetics between the POUR group and the non-POUR group.

**Table 2:** Differences in clinicopathological variables between surgical outcome groups

Factor (number [%] or median [IQR])	Postoperative urinary retention		*P* value
	No (*N* = 923)	Yes (*N* = 23)	
Age, years	68.3 (59.5, 74.6)	72.2 (69.3, 80.6)	0.012
Sex			0.510
Female	75 (8.1%)	1 (4.3%)	
Male	848 (91.9%)	22 (95.7%)	
Body mass index, kg/m^2^	23.2 (21.2, 25.0)	23.8 (21.8, 25.7)	0.320
Married			0.190
No	277 (30.0%)	4 (17.4%)	
Yes	646 (70.0%)	19 (82.6%)	
Benign prostate hypertrophy	156 (16.9%)	8 (34.8%)	0.025
Charlson comorbidity index score			0.780
≤ 2	658 (71.3%)	17 (73.9%)	
> 2	265 (28.7%)	6 (26.1%)	
American Society of Anesthesiologists physical status			0.120
< 3	549 (59.5%)	10 (43.5%)	
≥ 3	374 (40.5%)	13 (56.5%)	
Minimally invasive surgery	14 (1.5%)	0 (0.0%)	0.550
Bilateral repair	64 (6.9%)	2 (8.7%)	0.740
Spinal anesthesia	234 (25.4%)	5 (21.7%)	0.690
Volume of intraoperative fluid	400.0 (200.0, 500.0)	500.0 (250.0, 600.0)	0.040
Potency of anesthetics			
Short-acting	824 (89.3%)	21 (91.3%)	0.760
Intermediate-acting	531 (57.5%)	11 (47.8%)	0.350
Long-acting	308 (33.4%)	6 (26.1%)	0.460
Narcotic analgesics	743 (80.5%)	17 (73.9%)	0.430
Use of intraoperative vasopressors	343 (37.2%)	9 (39.1%)	0.850
Major complications	1 (0.1%)	0 (0.0%)	0.870
Length of hospital stay	3.0 (3.0, 3.0)	3.0 (3.0, 4.0)	0.008
Surgery duration (minutes)	69.0 (52.0, 86.0)	63.0 (54.0, 83.0)	0.950
Blood loss (ml)	0.0 (0.0, 0.0)	0.0 (0.0, 0.0)	0.120
Hernia repair done by general surgeons	581 (62.9%)	15 (65.2%)	0.820

Table 3 presents the multivariate adjusted model to predict the occurrence of POUR. Both increased age (OR 1.04, 95% CI 1.01–1.08; *P* = 0.049) and a greater volume of intraoperative fluid administered (OR 1.12 per 100-mL increase, 95% CI 1.01–1.27; *P* = 0.047) were significantly associated with the occurrence of POUR. A diagnosis of BHP (OR 2.12, 95% CI 0.86–5.22; *P* = 0.100) did not significantly predict POUR.

**Table 3:** Multivariate analysis to predict postoperative urinary retention

	Odds ratio	95% CI	*P* value
Age (per 1-year increase)	1.04	1.01–1.08	0.049
Benign prostate hypertrophy	2.12	0.86–5.22	0.100
Volume of intraoperative fluid (per 100-ml increase)	1.12	1.01–1.27	0.047

Furthermore, one adjusted linear regression analysis was conducted to predict the volume of intraoperative fluid administered (Table 4) according to age, sex, body mass index, CCI category, spinal anesthesia, use of intraoperative vasopressors, long-acting anesthetics, and blood loss. The results showed that the use of intraoperative vasopressors [coefficient = 101.22; 95% CI 66.25 to 136.19; *P* < 0.001] and blood loss (coefficient = 3.91; 95% CI 2.90 to 4.93; *P* < 0.001) were significantly associated with the volume of intraoperative fluid administered.

**Table 4:** Multivariate analysis to predict the volume of intraoperative fluid administered

Variable	Coefficients	95% CI		*P* value
		Lower limit	Upper limit	
Age (per 1-year increase)	0.67	−0.67	2.02	0.325
Male sex (ref: female)	71.24	−8.23	112.25	0.061
Body mass index (per one-unit increase)	3.20	−1.92	8.33	0.221
Charlson Comorbidity Index score > 2 (ref: ≤ 2)	−4.14	−41.75	33.46	0.829
Spinal anesthesia	−0.26	−66.23	65.71	0.994
Use of intraoperative vasopressors	101.22	66.25	136.19	< 0.001
Long-acting anesthetics	46.36	−14.6	107.32	0.136
Blood loss	3.91	2.90	4.93	< 0.001

## Discussion

POUR is one of the most common complications following IHR and can compromise recovery. Although POUR is often considered a minor complication by surgeons, it may have deleterious outcomes if not managed in a timely manner. The present study showed that POUR developed in 2.4% of the study population. Both increased age and a greater volume of intraoperative fluid administered (dose-dependent correlation) were the most significant risk factors for POUR. Notably, there were no associations of POUR with anesthetic type, anesthetic potency, or surgical method (minimally invasive versus open) in our study.

Among the aging population, it is common to have lower urinary tract symptoms and diseases, which are associated with decreased physical function, age-related illness, and medications to treat diseases outside the lower urinary tract (Ouslander 1997). Furthermore, detrusor underactivity, defined as decreased strength and/or duration of the voiding contraction, may deteriorate urination function, which can result in urinary retention and urinary tract infections (Drake et al. 2014). Previous studies have reported that increased patient age is associated with an increased risk of POUR following IHR (Blair et al. 2017; Patel et al. 2015). As it is impossible to reverse the aging process, surgical professionals should preoperatively evaluate lower urinary tract discomfort to relieve illness immediately and closely monitor the postoperative urination function, especially in elderly patients. Elderly individuals are more prone to urinary retention due to age-related changes and conditions. Contributing factors include an enlarged prostate in men, weakened bladder muscles, neurological disorders, medication side effects, urinary tract infections, pelvic organ prolapse in women, reduced mobility, and cognitive decline. These factors can hinder urine flow or impair bladder function.

In addition to the significant relationship between older age and POUR, our findings are consistent with previous studies in that the amount of intraoperative fluid administered in the operating room increases the risk of POUR (Broderick et al. 2022; Keita et al. 2005). It was hypothesized that fluid administration may contribute to overdistention of the bladder (Jackson et al. 2019). We found that both the use of intraoperative vasopressors and blood loss were two risk factors associated with the volume of intraoperative fluid administered. The main reason to administer vasopressors is the occurrence of intraoperative hypotensive events, which may result from unstable cardiovascular function, the extent of anesthesia, or blood loss. Strategies to provide an appropriate degree of anesthesia and diminish blood loss may reduce the volume of intraoperative fluid administered. However, past studies showed no relationship between the amount of perioperative fluid administered and POUR among inguinal hernia patients undergoing laparoscopic repair (*N* = 340; 8.2%) (Broderick et al. 2022; Lau et al. 2002; Sivasankaran et al. 2014). This inconsistency can be explained by the following reasons. First, the aforementioned papers only included laparoscopic IHR. Second, the sample sizes ranged from 72 to 350, with a higher rate of POUR than that observed in our study (2.4% versus 4.0–8.3%). Based on our more recent findings, we suggest that POUR after herniorrhaphy may be partially eliminated by limiting the amount of fluid given intraoperatively.

The association between increased intraoperative fluid volume and POUR can be attributed to several mechanisms:

1. Bladder overdistension: Excessive fluid administration can overstretch the bladder, impairing detrusor muscle contraction and leading to retention.
2. Autonomic dysfunction: Large fluid volumes may disrupt autonomic nervous system regulation, affecting bladder and urethral sphincter function.
3. Electrolyte imbalance: Improper fluid balance can cause electrolyte abnormalities, potentially interfering with neural control of bladder function.
4. Increased urine production: High fluid volumes can enhance renal blood flow and filtration, increasing urine output. If postoperative voiding is inadequate, this can result in bladder overdistension.
5. Inflammatory response: Surgical procedures and fluid administration may trigger inflammation affecting the lower urinary tract, potentially impacting bladder contractility.

These mechanisms highlight the importance of careful intraoperative fluid management in preventing POUR.

Furthermore, the dose and potency of anesthetics should be considered carefully to prevent hypotension, which should be managed with fluid resuscitation.

We found that the incidence rates of POUR under spinal and general anesthesia were 1.8% and 2.6%, respectively. Although general anesthesia had a higher rate of POUR than spinal anesthesia, the differences were not significant. In a review of 72 studies, Jensen and colleagues found that the incidence of POUR with regional anesthesia (150 in 6191 patients, 2.42%, 95% CI 2.04–2.81%) was lower than that with general anesthesia (344 in 11,471 patients, 3.00%, 95% CI: 2.69–3.31%) (Jensen et al. 2002). However, the potency of anesthetics, rather than the type of anesthesia, is considered the main cornerstone of the development of POUR (Blair et al. 2017; Petros et al. 1991). We included the potency of anesthetics in the analysis to adjust for potential confounding interactions and ensure that the findings were more informative.

This study has some limitations. First, given the nature of a retrospective study conducted in one institution, some bias may be present. The retrospective nature of our study limits our ability to establish causality. Furthermore, the majority of IHR in our study involved open repair, which may limit the generalizability of our findings to minimally invasive procedures. However, the sample size was nearly one thousand, and the perioperative parameters were retrieved from a research database integrated with the electric medical records. Furthermore, all IHR procedures were performed in an inpatient setting under the regulation of the Taiwan Healthcare System, and the patients are discharged the day after surgery. Thus, an early diagnosis of POUR can be achieved through more comprehensive consideration compared with an outpatient setting. Therefore, we consider our findings to be informative. Second, the majority of IHR in our study involved open repair. Some studies have reported that minimally invasive IHR leads to a higher incidence of POUR than the open method because of the proximity of dissection to the urinary bladder (Winslow et al. 2004; Koch et al. 2006). Third, outpatient IHR can reduce inpatient admissions and healthcare costs, whereas its choice is associated with clinical and socioeconomic factors.

Future prospective studies or randomized controlled trials could validate our findings and explore the efficacy of targeted interventions to reduce POUR risk, such as minimal invasive surgery, implementing protocols for judicious fluid administration in high-risk patients.

## Conclusions

As reflected by our findings and the results of other reports, POUR is a multifactorial disorder following IHR. In this series, we identified both increased patient age and a greater volume of intraoperative fluid administered as risk factors for POUR. Effective prevention of POUR may be accomplished by implementing strategies such as adopting routine preoperative assessments of lower urinary symptoms, avoiding intraoperative hypotensive events, and diminishing blood loss. Further research is necessary to validate these strategies.

## Data Availability

The data will be available if requested to the corresponding author.

## Data availability

No datasets were generated or analysed during the current study.
